# Medical Items

**Published:** 1893-01

**Authors:** 


### *MEDICAL ITEMS.*

THE JOURNAL extends a cordial greeting to all its readers and wishes them a happy and prosperous New Year.

---

DR. JOHN R. LEAMING, of New York, the well known author and teacher, died in December.

---

AT Union Point, Ga., December 22d, Dr. Benjamin F. Daniel was married to Miss Florence M. Householder. The happy couple have our best wishes.

---

DR. FRANK H. SIMS, formerly of Mobile, Ala., after spending several months abroad, has located in Atlanta. He will limit his practice to diseases of the eye, ear, nose and throat.

---

A ST. LOUIS physician reports that there are no less than twenty thousand persons in that city who habitually practice hypodermic injections on themselves, the great majority being women of the well-to-do classes.

---

DR. ROBERT E. MOSS has moved from Mexia, Texas, to San Antonio, where he will practice only diseases of the eye and ear hereafter. Dr. Moss spent the greater part of the summer working up his specialty in New York.

---

PROF. HARE considers ichthyol ʒi to ℥i of ointment the best local application for erysipelas. The affected part should first be washed with a mild solution of corrosive sublimate. Along with this local treatment he administers large doses of tincture of chloride of iron internally.

IN an editorial in the *Eclectic Medical Journal* the writer says: "I used to say to my students, " learn to keep a close mouth and look wise. No person can tell how big a fool you are if you keep your mouth shut."

To those who propose to follow the eclectic vagary this advice is doubtless very timely.

---

AT a meeting of the Board of Trustees on Wednesday, November 30th, 1892, Dr. G. E. de Schweinitz was, on the unanimous recommendation of the faculty, elected Clinical Professor of Ophthalmology in the Jefferson Medical College.

At the time of his election, Dr. de Schweinitz was Professor of Ophthalmology in the Philadelphia Polyclinic and Lecturer on Medical Ophthalmoscopy in the University of Pennsylvania.

---

THE committee on organization of the Pan-American Medical Congress will issue the preliminary announcement of the congress within a few weeks. This announcement will show that the organization has been perfected in almost every colony and county of the Western Hemisphere. The local medical societies in each of the constituent countries are made auxiliary to the congress, which will be held in Washington, D. C., September 5th, 6th, 7th, 8th, 1893.

Dr. Reed, of Cincinnati, Secretary-General of the Congress and Chairman of the Committee on Organization, announces that after extended correspondence between himself and Dr. Maragliano, of Genoa, General Secretary of the International Congress, the date of the Rome meeting has been finally and definitely set for September 24th of next year. This gives an interval of sixteen days between the Washington and the Rome meetings, during which an easy trip can be made from the former to the latter city. It is possible that a steamer may be chartered direct from New York to Rome.

### A NATIONAL QUARANTINE LAW.

President Harrison in his recent message to Congress strongly urges the importance of placing the entire quarantine system of this country under government control, and it now remains for our national legislators to formulate a new law that is easy, simple and effective. All State quarantine laws and regulation, should be properly changed and modified so as not to conflict with such general law in any way whatever. We cannot as a nation longer occupy a position of apparent indifference on this important subject with any degree of safety. Our one hope of security must come from the establishment of a thoroughly organized and equipped sanitary system under control of our Marine Hospital service, and this service should assume the entire responsibility of protecting our seaports against the introduction of contagious diseases of every description.—*Doctors' Weekly.*

---

A CORRESPONDENT informs us that female physicians are scarce everywhere except in the United States. There are only seventy in London, five in Edinburg, two in Dublin, thirty-five in Paris, one in Algiers, and two thousand in the United States. Has our correspondent not overlooked the fact that physicians are so numerous in the United States, with its sixty millions of people, that the proportion is vastly in excess in number of that of any other country ? This is the reason we have more female physicians here. It is because we have more of all kinds. Our system of licensing men and women to practice medicine in the United States is so lax that M. D.'s have become even more numerous, throughout the whole country than lawyers in great cities. As all our readers know, New York State, and some other of the States of the Union, have taken steps, by their law creating boards of medical examiners, which will, in another generation, lessen this excess in the number of physicians in the United States.—*Postgraduate.*

THE Association of Medical Colleges which met in Detroit on the 8th ult. says the *Western Medical Reporter*, during the meeting of the American Medical Association, promises to exert a most excellent influence on medical education. No college can be a member which does not require three years' attendance on sessions of six months' length. Two-thirds of the colleges belong to it and are living up to the requirements of the constitution. It is to be hoped that every college in the country will so amend its curriculum and extend its courses that it may join. The exchange of ideas and methods are necessarily intructive, and few men can avoid learning in such a gathering. Special training is required to equip a man for teaching in every branch of knowledge except medicine.

As several States have arbitrary laws forbidding the issuing of license to practice to graduates of schools which do not require attendance on three courses of lectures regardless of the applicant's fitness or ability, a school that gives less is a moral wrong. If State Licensing Boards would issue lists of the colleges whose diplomas are not recognized, the laggards in the race wou d be whipped up and forced to allow the sun of modern progress to penetrate their moss-covered shells. The Association of Medical Colleges is doing a good work, and every friend of higher medical education wishes it continued success and extended influence.

---

THE "American Text Book of Surgery" edited by Professors Keen and White of Philadelphia, which has only been issued a few months, is already a phenominal success. It has been adopted as a "Text Book" by forty-nine of our leading medical colleges and universities. Nearly five thousand copies have been placed in physicians' libraries, and every indication points to a sale of at least as many copies more in the next six months.

